# Effect of the Pitot Tube on Measurements in Supersonic Axisymmetric Underexpanded Microjets

**DOI:** 10.3390/mi10040235

**Published:** 2019-04-06

**Authors:** Sergey G. Mironov, Vladimir M. Aniskin, Tatiana A. Korotaeva, Ivan S. Tsyryulnikov

**Affiliations:** Khristianovich Institute of Theoretical and Applied Mechanics, Siberian Branch of Russian Academy of Sciences, Novosibirsk 630090, Russia; mironov@itam.nsc.ru (S.G.M.); korta@itam.nsc.ru (T.A.K.); tsivan@itam.nsc.ru (I.S.T.)

**Keywords:** supersonic microjets, Pitot tube

## Abstract

This paper describes the results of methodical investigations of the effect of the Pitot tube on measurements of gas-dynamic parameters of supersonic axisymmetric underexpanded real and model microjets. Particular attention is paid to distortions of Pitot pressure variations on the jet axis associated with the wave structure of the jet and to distortions of the supersonic core length. In experiments with model jets escaping from nozzles with diameters ranging from 0.52 to 1.06 mm into the low-pressure chamber, the measurements are performed by the Pitot tubes 0.05 to 2 mm in diameter. The results are analyzed together with the earlier obtained data for real microjets escaping from nozzles with diameters ranging from 10 to 340 µm where the parameters of real microjets were determined by the Pitot microtube 12 µm in diameter. Interaction of the Pitot tube with an unsteady jet in the laminar-turbulent transition region is investigated; the influence of this interaction on Pitot pressure measurements is determined, and a physical interpretation of this phenomenon is provided.

## 1. Introduction

The classification of channels in terms of their size, which was proposed in [1] and which is based on Knudsen numbers, implies that microchannels are those having the characteristic size from 10 to 200 µm. Taking into account this classification and extending it to micronozzles, we can assume that microjets are those escaping from nozzles with the characteristic size smaller than 200 µm (diameter for axisymmetric nozzles and height for plane and rectangular nozzles).

The measurement of flow parameters in gas microjets is a difficult diagnostic problem. Because of the small sizes of microjets, the use of a number of known methods for diagnosing gas flows is limited, e.g., shadowgraphy and Schlieren diagnostics, laser-induced fluorescence, particle image velocimetry, and hot-wire anemometry. The most suitable tool for microjets is the Pitot tube. However, the main problem in using this method is fabrication of Pitot tubes much smaller than the characteristic scale of microjet flows to ensure local measurements of jet parameters and to avoid jet flow distortions.

There are only a few investigations of supersonic microjet flows [2,3,4,5,6,7,8]. The most comprehensive study of supersonic microjet flows was performed in [7,8], where supersonic jets escaping from micronozzles 10 to 340 µm in diameter in the range of Reynolds numbers from 300 to 27,000 were considered. The authors [7] managed to fabricate a Pitot tube of 12 µm in diameter, which is the smallest sensor of this kind at the moment. The authors [7] also obtained pioneering data on the size of the gas-dynamic structure cell (barrel) and supersonic core length. It was found that the barrel size in supersonic microjets do not differ much from the barrel size in supersonic macrojets. However, it was demonstrated that the supersonic core length in microjets and macrojets is significantly different. The authors [8] performed experiments with the use of hot-wire anemometry for studying integral fluctuations of the mass flow rate of supersonic microjets. It was shown that the increase in the supersonic core length in microjets is associated with the laminar flow in the mixing layer of the jets, whereas the drastic decrease in the supersonic core length is caused by turbulization of the mixing layer of the jets.

The main parameter in investigations of supersonic jets is the jet pressure ratio (JPR), which is defined as the ratio of the static pressure at the nozzle exit to the ambient pressure. The JPR for microjets escaping into the atmosphere with the ambient pressure of about 1 atm is rigorously related to the Reynolds number via the pressure (and, hence, density) in the settling chamber, i.e., each Reynolds number corresponds to a certain JPR value. In the case of escaping of supersonic microjets into the ambient atmosphere [7] from nozzles 10–340 µm in diameter, the Reynolds numbers ranged from 300 to 27,000, with JPR changing from 1 to 4. However, the same Reynolds numbers can be reached by organizing a supersonic flow from a macro-sized nozzle into a low-pressure ambient space. Having one macro-sized nozzle and choosing appropriate values of the ambient pressure and the pressure in the settling chamber, one can simulate a supersonic flow of microjets escaping from micronozzles of different diameters into the atmosphere. Such macrojets can be called low-Reynolds-number jets or model microjets. A certain pair of numbers (Reynolds number and JPR) reached in the case of a jet escaping from the macronozzle to the low-pressure region always corresponds to a pair of such numbers for the microjet escaping from the micronozzle of a certain diameter into the atmosphere. The diameter of this nozzle is called the model diameter, because it is used as a basis for flow modeling in terms of the Reynolds number (for the corresponding JPR value). The possibility of modeling the structure of microjets (barrel size and supersonic core length) by using macrojets in terms of the Reynolds number calculated on the basis of the macronozzle diameter and gas parameters at the nozzle exit was discussed in [9,10,11]. It was also demonstrated there that the Reynolds number at which the laminar jet flow transforms to the turbulent state is identical for both real microjets and model microjets.

Modeling of microjets on the basis of macrojet studies offers a possibility of extending investigations of real microjets. In particular, a problem important for practice can be solved—determining the maximum possible diameter of the Pitot tube with respect to the nozzle diameter, which would ensure correct measurements of the wave structure and correct determination of the supersonic core length in microjets. For solving this problem, we studied supersonic jets escaping from nozzles 0.52, 0.72, and 1.06 mm in diameter into a low-pressure chamber. The results were analyzed together with the data of [7,8], where the parameters of real microjets escaping from nozzles with diameters ranging from 10 to 340 µm were determined by the Pitot microtube 12 µm in diameter. Moreover, it was of interest to study the influence of an unsteady axisymmetric underexpanded microjet flow in the laminar-turbulent transition region on results measured by the Pitot tube.

## 2. Experimental Equipment

**Figure 1:**
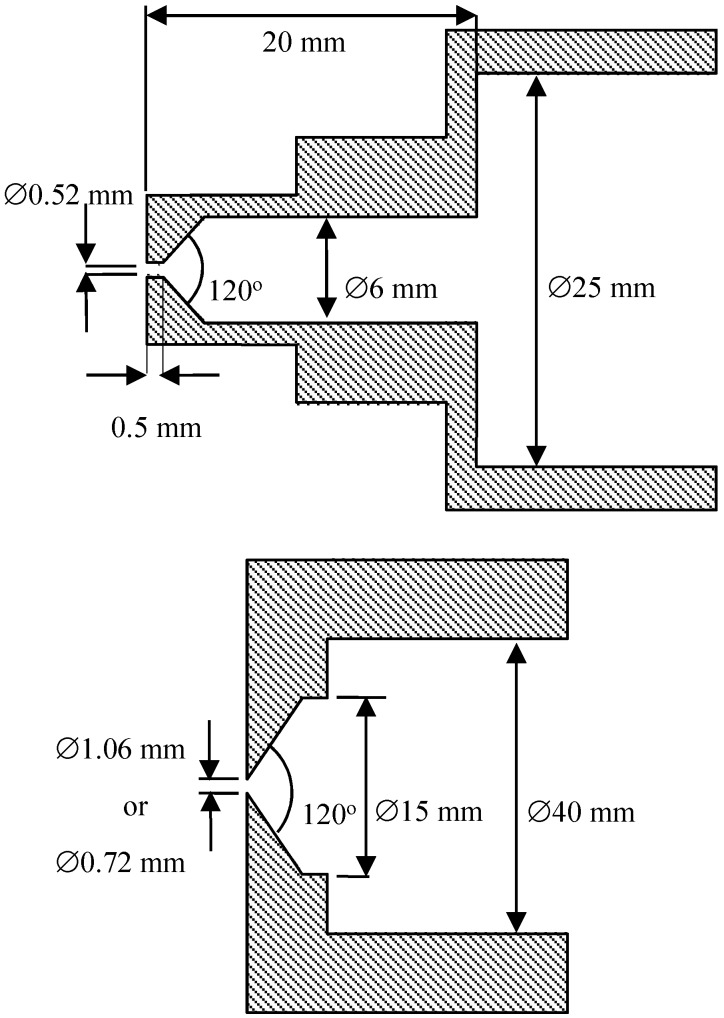
Nozzle scheme.

The experimental studies were performed for model air microjets escaping from sonic nozzles with diameters d = 0.52, 0.72, and 1.06 mm in a low-pressure jet setup. The nozzle scheme is shown in Figure 1. The nozzles had a different internal geometry.

The low-pressure jet setup is described in detail in [9] and was a sealed chamber evacuated by a vacuum pump with a nozzle system containing a sensor for measuring the stagnation pressure *P*_0_ of the jet being mounted in the chamber wall. Another pressure sensor was placed in the chamber for measuring the ambient pressure *P*_a_ of the medium to which the jet was exhausted. For example, for the jet escaping from the nozzle 1.06 mm in diameter to modeling the jet from the real size nozzle of 16.1 µm in diameter, the low-pressure ambient space *P*_a_ was set at 1500 Pa and *P*_0_ ranged from 3000 to 17,000 Pa (that corresponded with JPR from 1.06 to 6 and Reynolds number from 460 to 2600).

The Pitot tube was mounted on a traversing gear, which ensured the possibility of probe motion in the interval from 0 to 200 mm. The Pitot tubes used in the present study are listed in Table 1.

The data in Table 1 are the outer diameter of the Pitot tube *D*, its internal diameter *D*_int_, and characteristic relaxation time τ of pressure in the probe with this particular tube. The characteristic relaxation time τ was determined from the time dependence of the Pitot pressure *P*_0_′ in the case of rapid interruption/release of the jet. The Pitot tubes with the external diameters of 0.075 and 0.1 mm were made of glass, while the Pitot tubes of other diameters were made of stainless steel or copper.

The nozzle was mounted outside the low-pressure chamber and was equipped with microscrews for changing the nozzle position. With the use of these microscrews, the nozzle was mounted in such a way that the jet axis coincided with the line of Pitot tube motion. Their coincidence was monitored using transverse pressure distributions. The pressure was measured by Pitot tubes with external diameters of 0.075 and 0.1 mm and was performed at the points on the jet axis with allowance for the characteristic relaxation time. The pressure measurements by other Pitot tubes were performed with continuous motion of the tubes along the jet with a velocity of 0.25 mm/s. The velocity of Pitot tube motion was determined experimentally—it was chosen in such a way that the pressure distributions measured by the Pitot tube with the external diameter of 0.4 mm moving along the jet axis away from the nozzle and then back toward the nozzle were identical.

The pressures in the jet setup and in the Pitot tube were measured by TDM4-IV1 differential pressure probes (for pressures up to 0.4 bar) or TDM2-IV2 pressure probes (for pressures up to 1 bar). The electric signals from the pressure probes were digitized by a 12-bit analog to digital converter with frequency of 20 Hz.

The data determined in the experiments were the axial distributions of the Pitot pressure *P*_0_′ as functions of the nozzle diameter *d*, Pitot tube diameter *D*, and jet pressure ratio *n*. The JPR value is related to the pressure in the settling chamber by the formula 1/[1 + (γ − 1)/2]^γ/(γ−1)^, where γ is the ratio of specific heats of the gas in the jet. The flow at the supersonic jet axis transforms from supersonic to subsonic when *P*_0_′ reaches the value *P*_a_[1 + (γ − 1)/2]^γ/(γ−1)^, where *P*_a_ is the ambient pressure. The distance from the nozzle exit to the point where the supersonic flow transforms to the subsonic flow was determined as the supersonic core length *L*_C_. The jet setup structure allowed made it possible to establish and fix the pressure of the surrounding space *P_a_* when the jet flows into the chamber.

In addition, we also performed experiments recording the noise of the supersonic jet in the test section of the jet setup (both the free jet and the jet interacting with the Pitot tube). In particular, we measured the spectrum of acoustic oscillations in the ambient space around the jet. The spectral composition and the amplitude of the jet noise were correlated with the position of the frontal end of the Pitot tube and the value of the Pitot pressure *P*_0_′ as the Pitot tube was moved along the jet axis. The acoustic oscillations were detected by a PCB 132A31 piezoelectric sensor with a frequency range up to 500 kHz. The acoustic sensor of 3 mm in diameter was located at a distance of 10 mm from the jet axis and 27 mm from the nozzle. The axis of the direction indicatrix of the acoustic sensor was directed to the point on the jet axis located at a distance of 15 mm from the nozzle. The maximum level of acoustic oscillations of the supersonic jet was expected to be observed in the vicinity of this point.

## 3. Numerical Simulations

ANSYS Fluent commercial software (version 12) was used to simulate interaction of the Pitot tubes with outer diameters *D* = 0.4, 0.7, 1.1, and 2 mm with a supersonic underexpanded air jet, which escaped into the atmosphere from a nozzle with a diameter *d* = 0.5 and 1.06 mm at Reynolds numbers corresponding to modeling of a microjet escaping from a nozzle 16.1 μm in diameter and jet pressure ratio *n* = 2. Unsteady Reynolds-averaged Navier–Stokes equations were solved in an axisymmetric formulation with the use of the k-ω SST (shear stress transport) turbulence model. The solution procedure was performed by a “density-based” solver (term of the ANSYS Fluent software) and a second-order implicit scheme; convective fluxes were differentiated with the use of the Roe scheme. The computational domain included the settling chamber, the space around the jet, and the Pitot tube closed at the back side. The results of the computations were the unsteady and steady fields of Mach numbers and density around the jet and the Pitot tube, as well as the time-averaged pressure *P*_0_′ at the position of the frontal end of the tube at the jet axis at a distance equal to one half of the length of the first gas-dynamic cell of the jet structure. The computed data were compared with the measured results.

## 4. Results and Discussion

### 4.1. Effect of the Ratio of the Pitot Tube Diameter to the Nozzle Diameter

**Figure 2:**
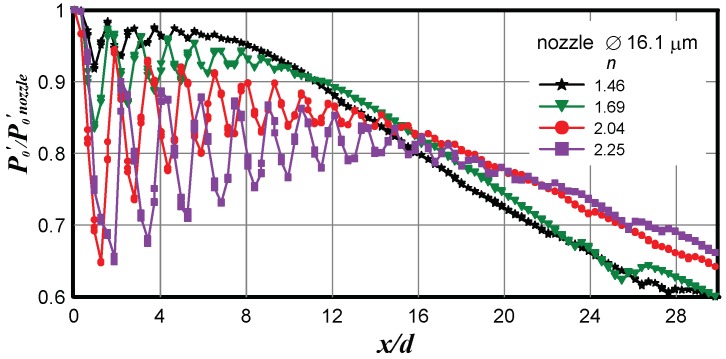
Axial pressure distribution measured by the Pitot tube in the jet escaping from the nozzle 16.1 µm in diameter.

As an example, Figure 2 shows a typical distribution of pressure along the jet axis. The data was taken from a supersonic (real) microjet escaping from the nozzle 16.1 µm in diameter by the Pitot tube 12 µm in diameter. The pressure curve has a quasi-periodic form associated with the shock wave structure of the jet. The peaks of the axial distribution of pressure were located at the ends of the jet barrels. The barrel length is one of the basic characteristics of supersonic jets. In turbulent jets, the barrel size and their transverse sizes from the first to the last barrel decrease owing to the evolution of the mixing layer of the jet. The parameters usually considered by most researchers are the length of the first barrel, the mean size of the barrels (mean sizes of the second, third, and fourth barrels), position and size of the Mach disk, and supersonic core length.

**Figure 3:**
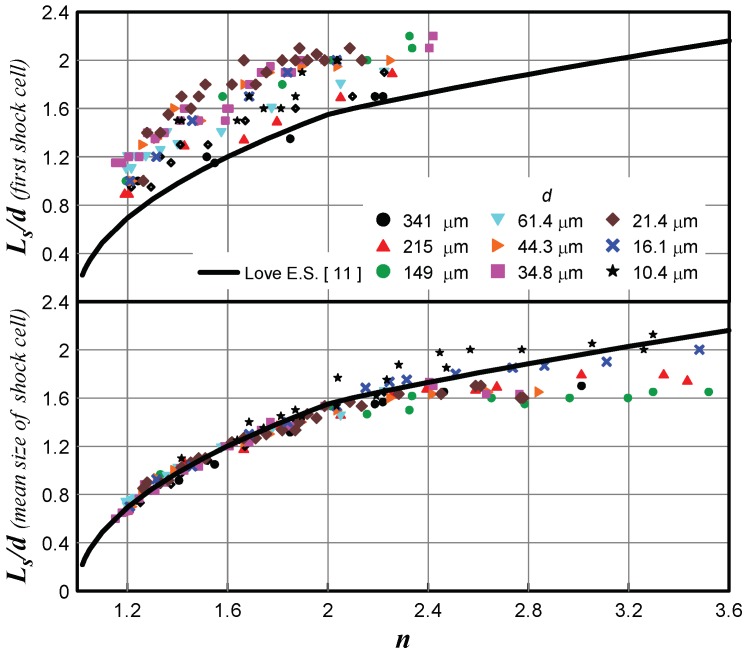
First barrel size and mean size of the barrels in real microjets.

The measurements of the first barrel length and the mean size of the barrels in real microjets performed by the Pitot tube 12 µm in diameter [7] showed that the mean barrel size was in excellent agreement with the first barrel size in macrojets [11] (except for those JPR values for which the mixing layer produces a significant effect). However, the first barrel size turned out to be greater than the first barrel size in macrojets almost for all jets, as shown in Figure 3, for all JPR values.

**Figure 4:**
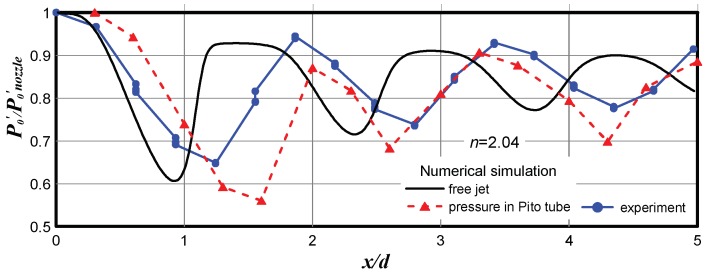
Comparison of the experimental and predicted data for a real microjet escaping from the nozzle 16.1 µm in diameter at *n* = 2.04.

To understand the reason for systematic overprediction of the first barrel size in microjets determined on the basis of the Pitot tube measurements, the following problem was solved. Exhaustion of a real supersonic microjet from the 16.1 µm nozzle into the ambient space (*P*_a_ = 1 atm, *P*_0_ = 3.86 atm) at *n* = 2.04 was considered. The Pitot tube was located on the axis of the jet at different distances *x*/*d*. The flow around the Pitot tube was calculated and the pressure inside the Pitot tube was determined. Additionally, the *P*_0_′ in the free jet was calculated. The results of numerical simulation and experimental data are compared in Figure 4.

It is seen that the calculated value of the pressure in the Pitot microtube located in the jet flow correlates fairly well with the experimental data. However, both these dependences were displaced with respect to the *P*_0_′ curve in the free jet approximately by 0.5 *d*. This shifting was induced by the detached shock wave formed on the Pitot microtube. As the Pitot tube measures the pressure behind the shock wave, we actually have a situation where *x*/*d* in Figure 4 corresponds to the Pitot tube position and *P*_0_′ corresponds to the shock wave position ahead of the Pitot tube. The distance between the Pitot tube and the shock wave ahead of the tube was not constant over the supersonic core of the jet; it varies periodically in proportion to the Mach number at the jet axis. The shift of the experimental data with respect to the *P*_0_′ curve for the free jet should be also affected by the ratio of the nozzle and Pitot tube diameters.

**Figure 5:**
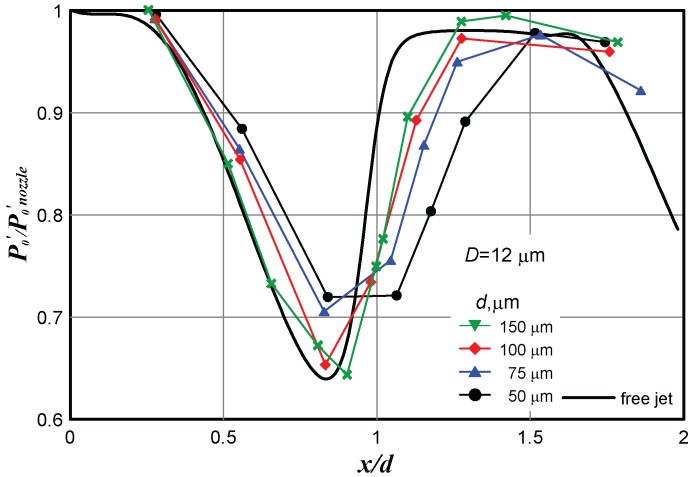
Calculated impingement of jets escaping from nozzles of different diameters onto the Pitot tube 12 µm in diameter.

Numerical calculations were performed for free jets escaping from micronozzles 50, 75, 100, and 150 µm in diameter at *n* = 1.8 and also for the same jets impinging onto the Pitot tube 12 µm in diameter. The results are plotted in Figure 5.

The distributions of *P*_0_′ calculated for jets having an identical JPR value but escaping from nozzles of different diameters almost coincide in the dimensionless coordinates. This distribution is shown by the solid curve in Figure 5. As the ratio *d*/*D* increases, the effect of the Pitot tube diameter on the accuracy of determining the position of the first barrel of the jet becomes less pronounced.

**Figure 6:**
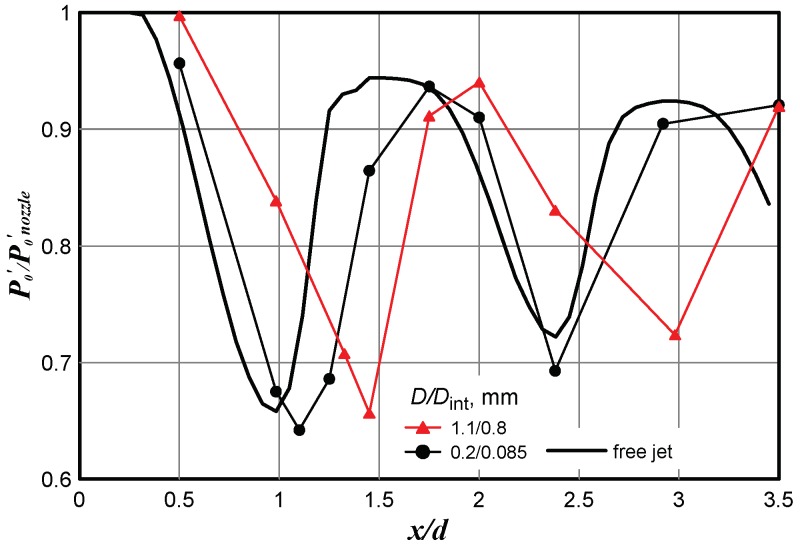
Calculation of the model microjet escaping from the nozzle 1.06 mm in diameter (modeling exhaustion from the nozzle 16.1 µm in diameter) and impinging onto the Pitot tubes of different diameters.

Similar results were obtained in model microjet calculations. In particular, Figure 6 shows the results of calculating the jet escaping from the nozzle 1.06 mm in diameter, modeling jet exhaustion from the nozzle 16.1 µm in diameter at *n* = 2. Figure 6 shows the calculated results for the free jet (solid line) and the calculated pressure in the Pitot tube located in the jet. The two sizes of the Pitot tube diameters were used in calculations—1.1 mm/0.8 mm and 0.2 mm/0.085 mm (outer/internal diameter). It is seen that the results were significantly affected by the shock wave position.

**Figure 7:**
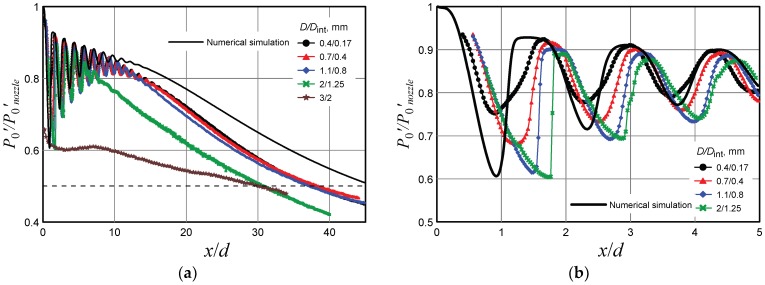
Pressure distribution in the jet escaping from the nozzle 1.06 mm in diameter (modeling exhaustion from the nozzle 16.1 µm in diameter). The plots in (**b**) are fragments from (**a**).

Figure 7 shows the *P*_0_′ distributions along the axis of the jet escaping from the nozzle with the diameter 1.06 mm and the jet pressure ratio *n* = 2. The pressure in the chamber was chosen in a way to model the flow from the nozzle 16.1 µm in diameter. The measurements were performed by the Pitot tubes with different diameters. The plots in Figure 7b are fragments from Figure 7a. The calculated data are shown by the solid black curve. The point of intersection of the curves with the dotted line corresponding to the pressure of the transition from the supersonic to subsonic flow shows the supersonic core length.

It is seen that the calculations predict a greater supersonic core length than that obtained in the experiments, as shown in Figure 7a. This difference is explained by the presence of a certain level of turbulence in the mixing layer of the real jet, as compared to the completely laminar model implied in the computations. The supersonic core lengths calculated from the experimental data obtained by the Pitot tubes with diameters up to 1.1/0.8 mm coincide with each other. For large Pitot tubes (2/1.25–3/2 mm), the supersonic core length was 20% smaller. The possibility of reliable determination of the supersonic core length by the Pitot tubes with comparatively large diameters was explained by the wide profile of the transverse pressure distribution in the jet cross section where the supersonic core length was determined. In this cross section, the maximum of the pressure distribution was fairly wide, and the tube diameter did not induce significant errors.

The pressure distribution near the nozzle exit was characterized by shifting of the experimental curves with respect to each other as the Pitot tube diameter increased, as shown in Figure 7b. As was demonstrated earlier, this shifting was determined by the detached shock wave. The greater the Pitot tube diameter, the greater the stand-off distance of the shock wave.

Though exhaustion of model microjets as a whole occurs in the continuum regime, there are local regions of reduced density in the jet, which correspond to the transitional flow regime from the viewpoint of the Knudsen number. In particular, the minimum density was observed in the middle of the first barrel, and it was in this region that the maximum influence of rarefaction on the results of Pitot tube measurements can be expected. It is worth mentioning that there was obvious disagreement in Figure 7b between the minimum of the pressure distribution in the first barrel of the jet obtained by the smallest Pitot tube (0.4/0.17 mm) and the calculated data.

At the point of the minimum pressure *P*_0_′ in the first barrel of the jet simulating exhaustion from the micronozzle with the diameter *d* = 16.1 µm, as shown in Figure 7b, the calculated gas density was *ρ* = 0.013 kg/m^3^, which corresponds to the molecular concentration *n* = 2.7 × 10^17^ molecules/cm^3^. Then the mean free path of molecules at this point was $\lambda =\frac{1}{\sqrt{2}n\sigma }$ = 2.5 × 10^−3^ cm. Here *σ* is the molecule collision cross section (*σ* ≈ 10^−15^ cm^2^). Thus, the local Knudsen number Kn directly calculated on the basis of the mean free path and Pitot tube size (*D* = 0.4 mm or *D*_int_ = 0.17 mm) is Kn = λ/*D* 0.06 or 0.15, respectively. These values correspond to the transitional regime of the flow around the Pitot tube (from continuum to free-molecular). According to [12], this is responsible for reduction of the Pitot tube readings, which was actually observed in experiments with these Pitot tube sizes. Moreover, because of gas rarefaction, the thickness of the shock wave ahead of the Pitot tube increases; as a consequence, the dependence *P*_0_′(*x*/*d*) becomes less steep, which was again observed in experiments.

Despite the visible shift of the experimental data with respect to the true distribution of *P*_0_′ at the jet axis, the mean size of the jet barrels was determined fairly accurately, which was confirmed by the experimental data in Figure 3. From this viewpoint, the Pitot tube provides reliable results, even if the Pitot tube diameter was comparable with the nozzle diameter.

**Figure 8:**
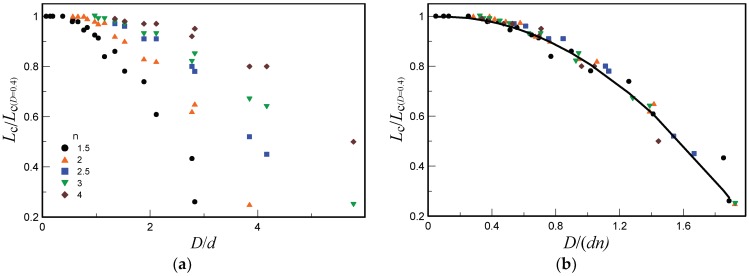
Normalized length of the supersonic core of the jet versus the ratio of the Pitot tube and nozzle diameters (**a**) and versus the ratio of the Pitot tube diameter *D* and nozzle diameter *d* multiplied by *n* (**b**).

Figure 8a shows the normalized length of the supersonic core of the jet as a function of the ratio of the outer diameter of the Pitot tube to the nozzle diameter *D*/*d* for several values of *n*. The supersonic core length was normalized to its value measured by the Pitot tube with the diameter *D* = 0.4 mm. It was seen that the normalized length of the supersonic core decreases as the ratio *D*/*d* increases. If the measured data shown in Figure 8a are plotted in the coordinates D/(dn), then all points fall on one decreasing curve, as is shown in Figure 8b.

In the region of the jet flow transition from the supersonic to subsonic state, the velocity profile in the jet becomes significantly expanded in the transverse direction, and the Pitot tube readings become less sensitive to the Pitot tube diameter. As a result, the condition for the Pitot tube diameter with respect to the nozzle diameter with 3% error is defined as *D*/(*dn*) ≤ 0.5.

### 4.2. Effect of Jet Flow Unsteadiness on Pitot Tube Measurements

**Figure 9:**
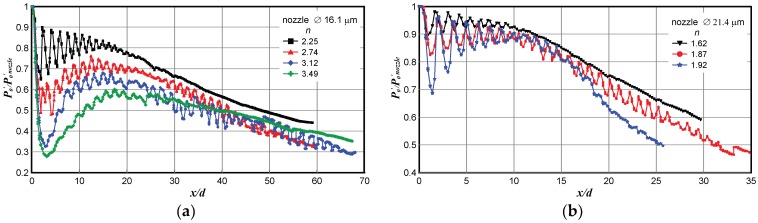
Pressure distributions in real microjets escaping from nozzles 21.4 (**a**) and 16.1 µm in diameter (**b**).

**Figure 10:**
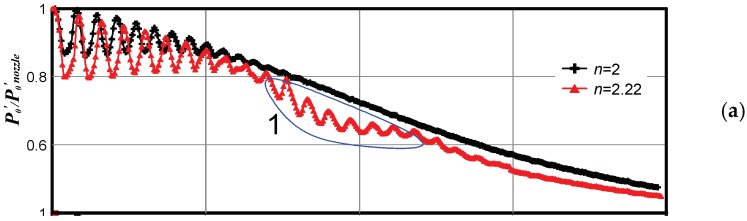

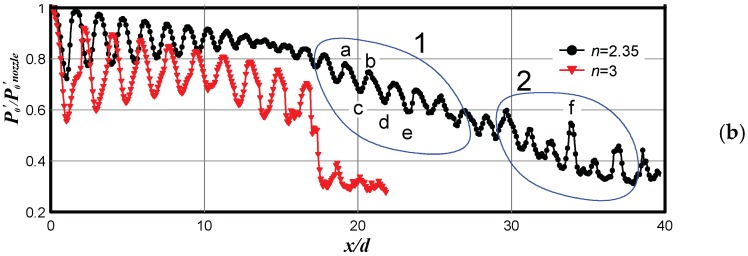
Pressure distribution in the model microjet escaping from the nozzle 1.06 mm in diameter (modeling exhaustion from the nozzle 16.1 µm in diameter). (**a**) *n* = 2 and 2.22, (**b**) *n* = 2.35 and 3.

The measurements of the Pitot pressure distributions *P*_0_′(*x*/*d*) on the jet axis at Reynolds numbers close to the conditions of the laminar-turbulent transition in the jet revealed significant steady fluctuations of the Pitot pressure. Such variations can be observed in both real, as shown in Figure 9, and model, as shown in Figure 10, microjets. In both real and model microjets, this effect was manifested for nozzle diameters 16–26 µm in a moderate range of JPR values.

As an example, Figure 9 shows the pressure distributions in real microjets escaping from the nozzles 21.4, as shown in Figure 9a, and 16.1 µm, as shown in Figure 9b, in diameter. All data in Figure 9 refer to the laminar regime of jet exhaustion, and minor pressure fluctuations were observed only in a small range of JPR values.

Figure 10 illustrates the emergence of pressure fluctuations with increasing JPR for the model microjet escaping from the nozzle with *d* = 1.06 mm (domain 1) and modeling jet exhaustion from the nozzle 16.1 µm in diameter. The measurements were performed by the Pitot tube with the diameter *D* = 0.4 mm. Exhaustion of the model microjet corresponds to the laminar flow regime at *n* = 2 and 2.2, to the transitional flow regime at *n* = 2.35, and to the turbulent flow regime at *n* = 3 (when the sharp drop-off of the pressure has occurred). Domain 2 in Figure 10b shows the measurements in the quasi-turbulent region of the jet, where random overshoots of the pressure *P*_0_′ were observed. It can be noted that the spatial period of pressure fluctuations at the jet axis in domain 1 coincides with the spatial period of pressure fluctuations on the cells of the wave structure of the jet near the nozzle.

**Figure 11:**
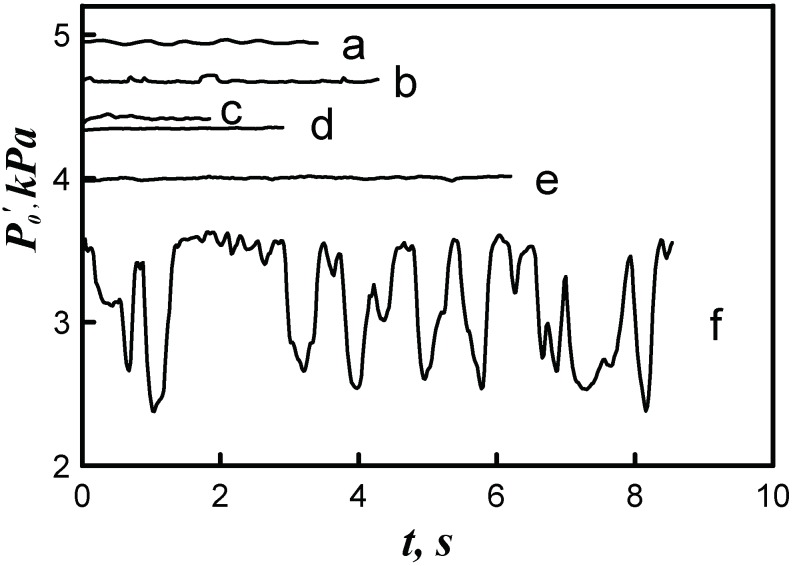
Time evolution of the pressure *P*_0_′ at the points (**a**–**f**) on the jet axis shown in Figure 10b.

The letters in Figure 10b indicate the points on the jet axis where the pressure evolution with time was measured. The measured results are shown in Figure 11. It is seen that the pressure at the measurement points in domain 1 were almost steady, whereas the pressure in domain 2 behaved randomly with time, which can be attributed to the transition of turbulent spots in the jet. Possibly, the frequency of passage of the turbulent spots (pressure jumps) was appreciably higher than that shown in the plot, but the pressure fluctuations in the experiments were averaged because of the finite characteristic time of pressure relaxation in the Pitot probe, and the sensor provided only an averaged pattern.

This behavior of pressure in domain 1 was typical for the emergence of acoustic feedback in a steady flow of supersonic underexpanded jets where screw instability modes B and C develop near the laminar-turbulent transition region [13]. The frequency of these modes was close to the frequency of acoustic waves in the ambient space, which, in turn, was equal to the velocity of sound in the ambient space divided by the doubled length of the gas-dynamic cells of the wave structure of the jet. The evolution and enhancement of these disturbances is the reason for global instability of underexpanded jets.

**Figure 12:**
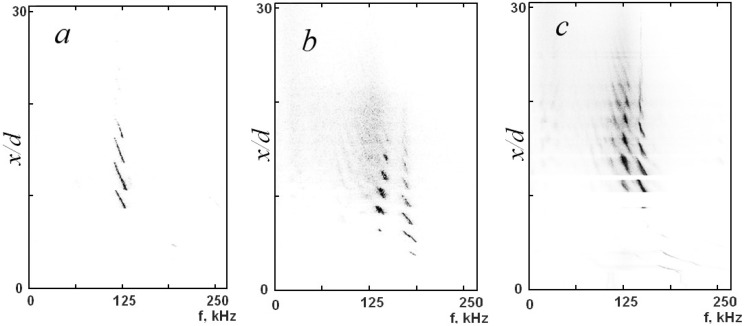
Spectra of acoustic oscillations generated by the underexpanded air jet in the plane of the normalized distance from the nozzle versus frequency for *n* = 1.75 (**a**), 2.5 (**b**), and 3 (**c**) for *d* = 0.72 mm and *D* = 0.4 mm.

In the plane of the normalized distance from the nozzle versus frequency, Figure 12 shows the spectra of acoustic oscillations detected by a piezoelectric sensor mounted near the jet in the case of motion of the Pitot tube with the diameter 0.4 mm for three values of *n*—1.75, 2.5, and 3. It is seen that there was one frequency of oscillations for *n* = 1.75, as shown in Figure 12a, and two frequencies for *n* = 2.5 and *n* = 3, as shown in Figure 12b,c. The frequency and amplitude of acoustic oscillations depends on the Pitot tube position on the jet axis.

The lower and upper frequencies of oscillations coincide with the frequencies of modes B and C of screw perturbations of the underexpanded jet, respectively. The periodicity of the emergence and vanishing of acoustic oscillations during the motion of the Pitot tube along the jet axis coincides with the periodicity of passing of the Pitot tube through the gas-dynamic cells of the wave structure of the jet. As the Pitot tube moves along the jet axis, Figure 12 in addition to variations of the amplitude of acoustic oscillations also shows periodic fluctuations of the frequency of these oscillations, which were noted in [14]. In the range *D* = 0.4–0.7 mm, a weak dependence of the amplitude of acoustic oscillations on the outer diameter of the Pitot tube was observed.

**Figure 13:**
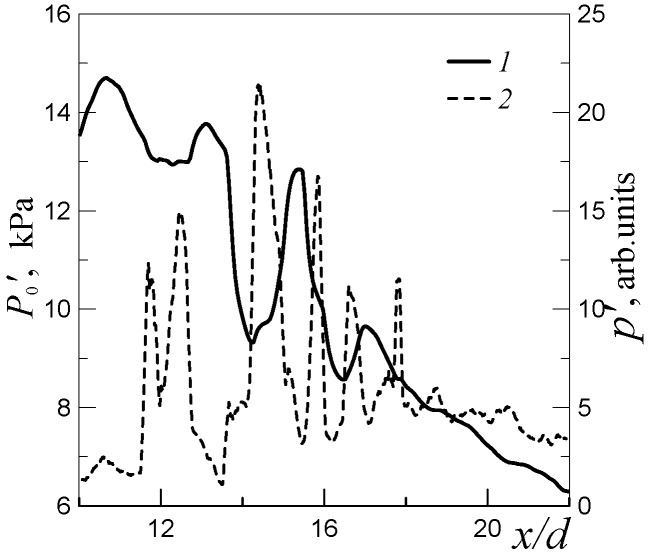
Pitot pressure *P*_0_′ (solid curve 1) and amplitude of acoustic oscillations *p*′ of mode B (dotted curve 2) versus the normalized distance from the nozzle; *d* = 0.72 mm, *D* = 0.4 mm, and *n* = 3.

As an example, Figure 13 shows the pressure *P*_0_′ and acoustic root-mean-square fluctuations of pressure *p*′ at the frequency of mode B in the ambient space as functions of the normalized distance from the nozzle. The pressure *P*_0_′ was measured by the Pitot tube with the diameter 0.4 mm. It is seen that the minimum points of *P*_0_′ coincide with the peaks of the acoustic oscillations of pressure in the ambient space of the jet.

The results obtained offer a simple physical explanation for the emergence of intense fluctuations of the pressure *P*_0_′ in the flow region close to the laminar-turbulent transition in the underexpanded jet. Intense fluctuations of screw modes of instability occur and develop in this region of the jet, leading to high-frequency motions of the gas-dynamic cells of the wave structure of the jet in the radial and azimuthal directions with respect to the jet axis with a spatial period equal to the length of two cells of the wave structure of the jet. Such motions were described in many publications dealing with instability of supersonic underexpanded macrojets, e.g., [13,15,16,17]. Interaction of the Pitot tube tap with an unsteady jet generates acoustic oscillations in the ambient medium, which propagate toward the nozzle exit, thus, creating feedback between the jet and the ambient medium, and can induce either enhancement or suppression of global instability of the jet. Enhancement or suppression depends on the phase of acoustic oscillations at the nozzle exit. In turn, the phase of acoustic oscillations depends on the Pitot tube position in the gas-dynamic cells of the wave structure of the jet. The feedback produces the minimum effects when the Pitot tube was located in a position where the phase at the nozzle exit corresponds to the node of acoustic oscillations. If the phase at the nozzle exit corresponds to the peak of acoustic oscillations, the maximum feedback effect is observed, resulting in jet flow failure, acceleration of mixing with the ambient medium, and decrease in *P*_0_′. These minimums and maximums were reached every time when the Pitot tube moved in one of the cells of the wave structure of the jet.

Thus, the motion of the Pitot tube along the jet axis generates periodic overshoots of acoustic oscillations, as shown in Figure 12. These acoustic oscillations provoke global instability of the underexpanded jet, which leads to acceleration of jet flow mixing with the ambient gas and to a decrease in *P*_0_′ with a spatial period equal to the length of the gas-dynamic cells of the wave structure of the jet. From this viewpoint, the amplitude of fluctuations of the pressure *P*_0_′ was actually overestimated because of the presence of the Pitot tube in the unsteady jet. Most probably, the true amplitude of Pitot pressure fluctuations would be appreciably lower if there were no Pitot tube in the jet.

## 5. Conclusions

The influence of the Pitot tube diameter on the pressure distribution *P*_0_′(*x*/*d*) and supersonic core length in an underexpanded jet was considered.

It was demonstrated that the experimental distributions of pressure along the axis of both real and model microjets were shifted with respect to the curves calculated for free microjets. It was found that this shifting was caused by the shock wave formed on the tube. As a result, there appears a significant error in determining the first barrel size on the basis of the pressure distribution. However, the shift of the experimental dependence does not affect the mean barrel sizes—they are determined exactly.

Though exhaustion of model jets as a whole occurs in the continuum regime, there were local regions of reduced density in the jet, which correspond to the transitional flow regime from the viewpoint of the Knudsen number, leading to significant distortions of the pressure distributions in the jet in these regions.

The Pitot tube diameter has a minor effect on determining the supersonic core length, which was explained by the wide profile of the transverse pressure distribution in the jet cross section where the supersonic core length was determined. In this cross section, the maximum of the pressure distribution was fairly wide, and the tube diameter does not induce significant errors. It was shown that acceptable accuracy of measurements of the supersonic core length can be provided if the inequality *D*/(*dn*) ≤ 0.5 is satisfied.

The presence of the Pitot tube in the jet in the region of the laminar-turbulent transition was found to produce a significant effect on the results of *P*_0_′(*x*/*d*) measurements in the jet flow. A physical mechanism of the emergence of intense fluctuations of the Pitot pressure *P*_0_′ on the underexpanded jet axis near the laminar-turbulent transition was proposed. The relationship between these fluctuations and interaction of the unsteady jet with the Pitot tube tap was revealed.

## Figures and Tables

**Table 1:** Pitot tube size and relaxation time.

*D*, mm	*D*_int_, mm	τ, s
0.075	0.05	100
0.1	0.075	17
0.4	0.17	0.33
0.6	0.3	0.06
0.7	0.4	0.02
1.1	0.8	<0.01
2	1.5	<0.01
3	2	<0.01
